# Molecular detection of medically relevant *Sporothrix* species in roadkilled wildlife in the Brazilian Atlantic forest

**DOI:** 10.1007/s11046-026-01067-4

**Published:** 2026-03-12

**Authors:** Steffanie Skau Amadei, Julia Campos, Andressa Maria Rorato Nascimento de Matos, Keity Aparecida Speçato, Eloiza Teles Caldart, Ana Paula Frederico Rodrigues Loureiro Bracarense, Ferry Hagen, Zoilo Pires de Camargo, Anderson Messias Rodrigues

**Affiliations:** 1 Integrated Program in Neuroscience, McGill University, Montreal, Canada; 2 School of Medicine and Life Sciences, Pontifical Catholic University of Paraná, Curitiba, Brazil; 3 Department of Preventive Veterinary Medicine, State University of Londrina, Londrina, Brazil; 4 Department of Medical Microbiology, University Medical Center Utrecht, Utrecht, The Netherlands; 5 Westerdijk Fungal Biodiversity Institute, Utrecht, The Netherlands; 6 Laboratory of Emerging Fungal Pathogens, Department of Microbiology, Immunology, and Parasitology, Discipline of Cellular Biology, Federal University of São Paulo (UNIFESP), São Paulo, 04023062 Brazil; 7 National Institute of Science and Technology in Human Pathogenic Fungi, São Paulo, Brazil

**Keywords:** Sporotrichosis, Roadkill, *Sporothrix brasiliensis*, *Sporothrix schenckii*, *Sporothrix globosa*, Wildlife

## Abstract

The rapid expansion of zoonotic sporotrichosis in South America necessitates innovative surveillance strategies to identify natural ecological niches. Roadkill provides a unique, underutilized opportunity to monitor *Sporothrix* circulation within human-impacted landscapes. We conducted a molecular survey via a triplex probe quantitative real-time PCR (qPCR) assay targeting pathogenic *Sporothrix* species in 81 roadkilled vertebrates (mammals, birds, and reptiles) collected along highways BR-376 and PR-445 traversing the Atlantic Forest in Paraná, Brazil (2017–2023). Genomic DNA from visceral organs (heart, liver, lung, and spleen) was screened for fungal DNA. *Sporothrix* DNA was detected in 13.6% (11/81) of the samples. *Sporothrix schenckii* predominated, identified in wild mammals (*Leopardus guttulus*, *Didelphis albiventris*, and *Lepus europaeus*) and diverse birds (*Colaptes melanochloros*, *Piaya cayana*, and *Selenidera maculirostris*), indicating systemic exposure. Strikingly, *S. brasiliensis* was detected in avian hosts (*Columbina picui*, *Crypturellus tataupa*), challenging the thermal-exclusion hypothesis and implicating birds as potential aerial vectors. Furthermore, *S. globosa* was found in *Dasyprocta* spp. and was co-detected with *S. brasiliensis* in *Columbina picui*. Notably, a reptile (*Oxyrhopus* spp.) was positive for *S. globosa* and *S. schenckii*, expanding the known host spectrum, potentially via trophic transmission. Although distinguishing transient DNA carriage from active infection requires histopathological validation, these findings suggest that wildlife in fragmented corridors may function as reservoirs, biological amplifiers, or mechanical vectors rather than incidental hosts, reinforcing the blurring boundaries between sylvatic and synanthropic transmission cycles. In this context, integrating roadkill biosurveillance into a One Health framework is vital for tracking environmental pathogen loads and anticipating zoonotic spillover.

**Supplementary Information:**

The online version contains supplementary material available at 10.1007/s11046-026-01067-4.

## Introduction

Every second, approximately 15 wild animals are killed on Brazilian roads, totaling an estimated 1.3 million fatalities daily and over 475 million annually, which threatens biodiversity [1]. However, roadkill remains an underutilized resource for the surveillance of emerging zoonoses [2]. Roadkill samples provide valuable data for zoonotic surveillance, early detection, risk factor identification, mitigation strategy development, and investigations into pathogen ecological niches [3, 4].

*Sporothrix* (*Ophiostomatales*) encompasses thermally dimorphic fungi that cause sporotrichosis, a subcutaneous mycosis with increasing zoonotic recognition [5]. In Brazil, *Sporothrix brasiliensis*, *S. schenckii*, and *S. globosa* are of public health and veterinary significance [6]. *Sporothrix brasiliensis* is the primary agent of cat-transmitted sporotrichosis (CTS), driving outbreaks across Brazil and neighboring countries [7]; this contrasts with the global sapronotic transmission pattern associated with *S. schenckii* and *S. globosa* [6]. Brazil’s CTS-driven scenario focused epidemiological efforts on domestic cats (*Felis catus*), leaving the role of neotropical wildlife as reservoirs, mechanical vectors, or spillover hosts as critical gaps in the ecological understanding of *Sporothrix* [8].

To address these gaps, we employed real-time quantitative PCR (qPCR), a well-established, sensitive, and precise method for *Sporothrix* DNA detection [9]. The successful application of qPCR in roadkill studies for detecting bacteria [10], viruses [11], and parasitic agents [12] has reinforced its utility for pathogen surveillance. *Sporothrix* DNA was surveyed from roadkill collected along highways traversing the Atlantic Forest in Paraná, Brazil, a region with frequent interactions between wildlife, domestic animals, and humans. Our findings suggest that emerging *Sporothrix* species have active sylvatic circulation and broader host distributions, providing novel insight into the hidden role of roadkill in sporotrichosis ecoepidemiology.

## Materials and Methods

### Study Area and Biological Sampling

**Fig. 1:**
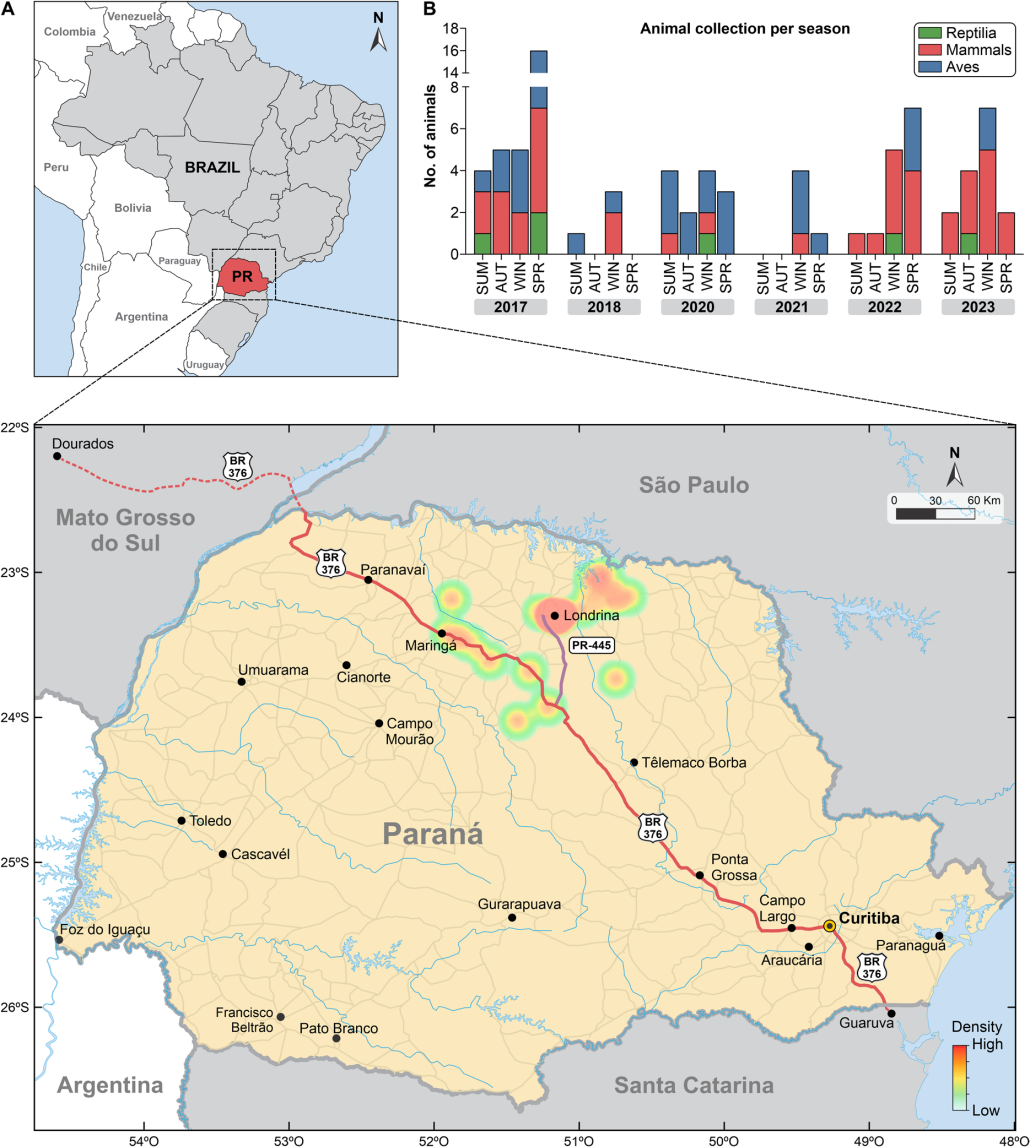
**A** Map of the study area in Paraná, Brazil, indicating the locations where 81 roadkilled animals were collected between 2017 and 2023. The sampling points are distributed along highways BR-376 and PR-445. **B** The collected fauna included mammals (*n* = 39), birds (*n* = 36), and reptiles (*n* = 6), and are detailed in Supplementary Table 1

This study was conducted along the BR-376 (approx. 530 km through biodiverse forests/grasslands) and PR-445 (approx. 150 km through forest fragments/agricultural lands) highways in Paraná, Brazil, within the Atlantic Forest biome and the Campos Gerais region [13, 14] (Fig. 1).

The collection of roadkill involved weekly active surveys and notifications from law enforcement. Carcasses were retrieved within 24 h of collision and not eviscerated. Species identification and tissue collection followed the guidelines of Caldart et al*.* [15] under Brazilian wildlife research regulations (Ethics Committee on Animal Use, State University of Londrina, protocol no. 30/2017; SISBIO license no. 55384–1).

### DNA Extraction and Quality Control

Genomic DNA was isolated from the collected tissue samples using the PureLink Genomic DNA Mini Kit (Invitrogen, Carlsbad, CA, USA) following the manufacturer’s instructions. The concentration and purity of the extracted DNA were evaluated via a NanoDrop 2000 spectrophotometer (Thermo Fisher Scientific, Wilmington, DE, USA). To confirm DNA integrity and exclude PCR inhibition, all samples were subjected to internal quality-control PCR targeting conserved vertebrate 12S and 16S rRNA sequences, as previously described [16]. Only samples in which vertebrate DNA was amplified were retained for pathogen screening.

### *Molecular Detection of Sporothrix* spp. via* qPCR*

Samples were screened for medically relevant *Sporothrix* DNA via a triplex probe quantitative real-time PCR (qPCR) assay targeting β-tubulin polymorphisms to simultaneously detect and differentiate *S. brasiliensis*, *S. schenckii*, and *S. globosa* [9]. Reactions were performed in 20 µL containing 10 µL of TaqMan Genotyping Master Mix (Thermo Fisher Scientific), 900 nM of each primer Sporo-F and Sporo-R (Integrated DNA Technologies, USA), 250 nM of each species-specific TaqMan hydrolysis probe (Sbra-FAM, Ssch-NED, and Sglo-VIC; Thermo Fisher Scientific), and 5 µL of DNA template. Amplification was run on a StepOnePlus instrument (95 °C for 10 min; 45 cycles of 95 °C for 15 s and 60 °C for 1 min). The samples were considered positive when they presented an exponential amplification curve within the validated Cq detection range [9]. Each run included no-template and species-specific positive controls (Ss54 for *S. brasiliensis*, Ss126 for *S. schenckii*, and Ss06 for *S. globosa*) to confirm the reliability of the assay.

Amplicons generated from the Sporo-F and Sporo-R primers were purified via the Wizard SV Gel and PCR Clean-Up System (Promega, Madison, WI, USA). To improve the quality of the sequencing data (Phred ≥ 30), the β-tubulin amplicons were subjected to control sequencing in two reactions. A BigDye Terminator v3.1 Cycle Sequencing Kit from Applied Biosystems was used for Sanger sequencing, and a SeqStudio Genetic Analyzer System was used to analyze the sequences (Applied Biosystems). Nucleotide sequences obtained from the β-tubulin amplicons were edited and assembled into consensus sequences using the CAP Contig Assembly Program implemented in BioEdit v. 7.0.9.0 [17].

### Phylogenetic Analysis

To contextualize the roadkill-derived sequences, homologous reference sequences of *Sporothrix* spp. were retrieved from the NCBI GenBank database [18–21]. Phylogenetic analysis was performed using the BioNumerics software v.7.6 (Applied Maths, Sint-Martens-Latem, Belgium). Multiple sequence alignment was calculated using the fast algorithm with the following settings: an open gap cost of 100% and a unit gap cost of 0%. The fast algorithm parameters were defined with a minimum match sequence of 2 and a maximum number of gaps of 99. Similarity matrices were calculated on the basis of multiple alignment. The default cost table was used, with no correction applied for evolutionary distance. Cluster analysis was performed using the unweighted pair group method with arithmetic mean (UPGMA) method [22]. The robustness of the tree topology was evaluated via bootstrap analysis with 1,000 simulations [23]. In addition, the best-fit nucleotide substitution model was selected on the basis of the lowest Bayesian information criterion (BIC) score, implemented in MEGA11 [24]. The analysis indicated that the Kimura 2-parameter model with a discrete gamma distribution and evolutionary invariant sites (K2 + G + I) provided the optimal fit for the data. The Gamma-shaped parameter was estimated at 0.43, and the fraction of invariant sites was 25.0%. Phylogenetic relationships were also inferred using the maximum likelihood (ML) method, using a bootstrap analysis with 1,000 replications [23]. The initial tree for the heuristic search was obtained automatically by applying the neighbor-joining and BioNJ algorithms to a matrix of pairwise distances estimated using the maximum composite likelihood (MCL) approach. The final dataset included 69 nucleotide sequences with a total of 193 positions, following the complete deletion of all positions containing gaps and missing data.

### Genetic Diversity and Haplotype Analysis

To elucidate the underlying genetic structure and reduce dataset dimensionality, principal component analysis (PCA) and multidimensional scaling (MDS) were employed. PCA and MDS utilized pairwise distance matrices in BioNumerics v.7.6 to identify the primary sources of variance, projecting the sequences into a three-dimensional coordinate space to visualize grouping behaviors and separation between taxa [25].

Complementary clustering and topology-based methods were applied in BioNumerics v.7.6 to further characterize sequence relationships. Minimum spanning trees (MSTs) were constructed to map the shortest evolutionary pathways between sequence types, illustrating the connectivity and distance between genotypes [26]. Additionally, self-organizing maps (SOMs) were generated via an unsupervised artificial neural network algorithm [27] to classify the entries on the basis of sequence similarity without a priori taxonomic assumptions. The SOM dimensions were optimized on the basis of sample size (determined by the heuristic formula $5.\sqrt{n}$) [28], with the resulting spatial arrangement reflecting the degree of genetic relatedness among the studied populations [29].

### Genetic Diversity and Haplotype Analysis

To assess the genetic variability of the identified *Sporothrix* populations, standard diversity indices were calculated via DnaSP software v.6.12 [30]. These metrics included the number of haplotypes (*h*), haplotype diversity (*Hd*), and nucleotide diversity (*pi*). To visualize the genealogical relationships and intraspecific divergence among the sequences, a haplotype network was constructed via the median-joining (MJ) algorithm implemented in Network software (Fluxus Engineering) [31].

### Statistical Analysis

Epidemiological variables were tabulated and analyzed to identify factors associated with *Sporothrix* spp. detection. Categorical variables (taxonomic class, IUCN status, road type, season, proximity to urban centers, and interaction with domestic animals) were summarized as absolute and relative frequencies. Associations with qPCR positivity were tested via Pearson’s chi-square test (*χ*^2^) or Fisher’s exact test when the expected counts were < 5. All analyses were two-tailed, with statistical significance set at *p* < 0.05, and performed in GraphPad Prism v9.0.

## Results

Between 2017 and 2023, 81 roadkill animals were collected: 39 mammals (48.15%), 36 birds (44.44%), and 6 reptiles (7.41%) (Fig. 1). A total of 178 tissue samples from the heart, liver, lungs, and spleen were collected (Supplementary Table 1). Medically relevant *Sporothrix* DNA was detected in 11 specimens (13.58%) across all three vertebrate classes (Table 1). Sanger sequencing provided orthogonal verification of the qPCR-positive samples (Supplementary Table 2). The tissues most frequently positive were the heart (*n* = 6) and liver (*n* = 5), suggesting potential primary infection or reservoir sites.

**Table 1:** Detection of *Sporothrix* spp. DNA in road-killed wildlife from Paraná, Brazil (2017–2023)

Class	Species	Common name	Coordinates (Lat./Long.)		Road	Season/Year	Tissue	qPCR (Cq)*		
								*S. brasiliensis*	*S. schenckii*	*S. globosa*
Aves	*Colaptes melanochloros*	Green-barred woodpecker	23°36′57″S	51°18′36″W	BR-376	Spring/2017	Heart	N/D	31,07	N/D
	*Columbina picui*	Picui ground dove	23°43′3″S	51°23′47″W	BR-376	Spring/2017	Liver	42,78	N/D	18,71
	*Crypturellus tataupa*	Tataupa tinamou	23°30′38″S	51°14′3″W	PR-538	Winter/2017	Heart	41,54	N/D	N/D
	*Piaya cayana*	Squirrel cuckoo	23°28′0.216′′S	51°8′2.202″W	PR-445	Autumn/2020	Liver	N/D	16,99	N/D
	*Selenidera maculirostris*	Spot-billed Toucanet	23°14′46″S	51°56′22″W	Rural	Summer/2018	Liver	N/D	26,07	N/D
Mammalia	*Dasyprocta* spp.	Agoutis	23°20′47.0″S	51°08′32.9″W	Urban	Autumn/2023	Liver	N/D	N/D	42,09
	*Didelphis albiventris*	White-eared opossum	23°21′16.6″S	51°11′54.3″W	Rural	Winter/2023	Heart	N/D	12,15	N/D
	*Leopardus guttulus*	Southern tiger cat	23°28′47′3″S	51°7′57.8″W	PR-445	Summer/2020	Heart	N/D	12,11	N/D
	*Lepus europaeus*	European hare	23°18′49.9″S	51°12′53.4″W	PR-445	Spring/2022	Heart	N/D	35,38	N/D
	*Lepus europaeus*	European hare	23°18′12″S	51°10′15″W	Urban	Summer/2017	Heart	N/D	41,00	N/D
Reptilia	*Oxyrhopus* spp.	False coral snake	N/A	N/A	Urban	Winter/2020	Liver	N/D	18,20	23,04

^*^Cq, quantification cycle; N/D, not detected; N/A, not available

Statistical analysis revealed that qPCR positivity was associated with road type (*χ*^2^(3) = 9.640, *p* = 0.0219) and interaction with domestic animals (Fisher’s exact test, *p* = 0.0322) but not with other key variables, including taxonomic class (*χ*^2^(2) = 3.000, *p* = 0.2231), IUCN conservation status (*χ*^2^(1) = 1.000, *p* = 0.3173), season (*χ*^2^(3) = 4.000, *p* = 0.2615), or proximity to an urban center (*χ*^2^(1) = 1.000, *p* = 0.3173) (Supplementary Table 3).

The species most frequently detected was *S. schenckii*, which was found in eight samples from seven roadkilled animals, including the green-barred woodpecker (*Colaptes melanochloros*; heart, Cq 31.07), the squirrel cuckoo (*Piaya cayana*; liver, Cq 16.99), the spot-billed toucanet (*Selenidera maculirostris*; liver, Cq 26.07), two European hares (*Lepus europaeus*; hearts, Cqs 35.38 and 41.00), the southern tiger cat (*Leopardus guttulus*; heart, Cq 12.11), the white-eared opossum (*Didelphis albiventris*; heart, Cq 12.15), and the false coral snake (*Oxyrhopus* spp.; liver, Cq 18.20) (Table 1).

*Sporothrix brasiliensis* was detected in two bird species, the picui ground dove (*Columbina picui*; liver, Cq 42.78) and the tataupa tinamou (*Crypturellus tataupa*; heart Cq 41.54). *Sporothrix globosa* was identified in the agouti (*Dasyprocta* spp.; liver, Cq 42.09), the picui ground dove (*Columbina picui*; liver, Cq 18.71), and the false coral snake (*Oxyrhopus* spp.; liver, Cq 23.04). Notably, evidence of coinfection was observed in two cases in which the picui ground dove carried both *S. brasiliensis* and *S. globosa* DNA, whereas the false coral snake harbored *S. schenckii* and *S. globosa* DNA (Table 1).

**Fig. 2:**
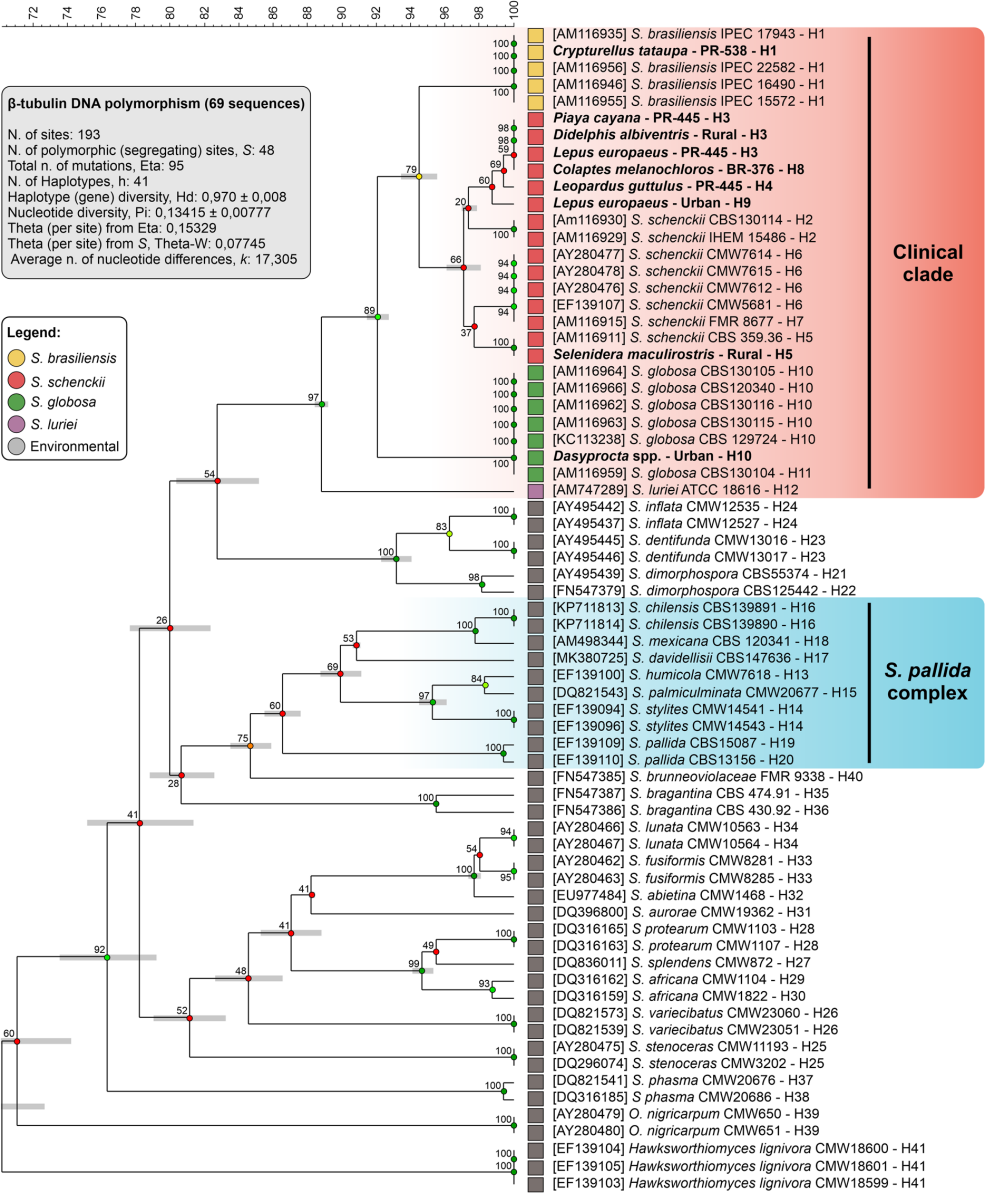
UPGMA reconstruction of *Sporothrix* species recovered from roadkill wildlife on the basis of partial β-tubulin (*BT2*) gene sequences. The dendrogram was generated via BioNumerics v.7.6 (Applied Maths). Multiple sequence alignment was performed via the fast algorithm (open gap cost: 100%; unit gap cost: 0%; minimum match sequence: 2; maximum gaps: 99). Cluster analysis was performed via the unweighted pair group method with arithmetic mean (UPGMA), which is based on similarity matrices with no correction for evolutionary distance. The topology confirms that roadkill-derived sequences (highlighted) cluster strictly within the clinical clade (*S. brasiliensis*, *S. schenckii*, and *S. globosa*). Branch support values derived from 1,000 bootstrap simulations are indicated at the nodes. The GenBank accession codes and haplotype (H) numbers are close to each taxon. Although 11 samples were positive by multiplex qPCR, only nine sequences were represented in the phylogenetic tree because two cases involved coinfections (*S. schenckii*/*S. globosa* and *S. brasiliensis*/*S. globosa*; Table 1), which generated mixed Sanger chromatograms with ambiguous polymorphic sites, precluding reliable sequence assembly and phylogenetic inference (Supplementary Table 2)

The analysis of the *BT2* gene fragment (69 sequences, 193 aligned sites) exhibited high genetic and haplotype variability, resolving 41 distinct haplotypes. Comprehensive polymorphism and nucleotide diversity metrics, indicating a substantial accumulation of genetic variation and divergent evolutionary history among the analyzed taxa, are summarized in Fig. 2 (inset) and Supplementary Table 4. The distribution of haplotypes was heterogeneous, with the majority being unique or shared by few individuals, suggesting active diversification. Notably, the roadkill-derived sequences were not randomly distributed but were consistently associated with specific, well-established genotypes described for the members of the clinical clade.

Phylogenetic reconstruction and distance-based analyses confirmed that all amplicon sequences obtained from the roadkill samples fell strictly within the medically relevant clinical clade. The sequences clustered robustly with reference strains of *S. brasiliensis* (IPEC 16490; 100% ± 0.0%), *S. schenckii* (CBS 359.36; 97.1% ± 0.91%), and *S. globosa* (CBS 120340; 100% ± 0.0%) (Fig. 2), corroborating the species identification previously obtained via qPCR. This phylogenetic placement provides definitive evidence that the detected DNA belongs to pathogenic *Sporothrix* species and rules out the presence of environmental contaminants, nonpathogenic *Sporothrix* or *Ophiostoma* relatives in the positive samples (Fig. 2).

The median-joining haplotype network further elucidated the genealogical relationships, showing that wildlife sequences often shared haplotypes or were immediate neighbors to clinical and veterinary isolates described in previous studies. For example, *S. brasiliensis* sequences from the tataupa tinamou (*Crypturellus tataupa*) and picui ground dove (*Columbina picui*) clustered within the dominant *S. brasiliensis* haplogroup (H1), which is indistinguishable from known cat-transmitted strains (*e.g.*, IPEC 16490 and IPEC 17943). Similarly, *S. schenckii* sequences from the squirrel cuckoo (*Piaya cayana*) and white-eared opossum (*Didelphis albiventris*) grouped in H3, demonstrating close genetic affinity with established lineages in H4 (CBS 359.36) and H5 (CMW 7614) (Supplementary Table 4).

**Fig. 3:**
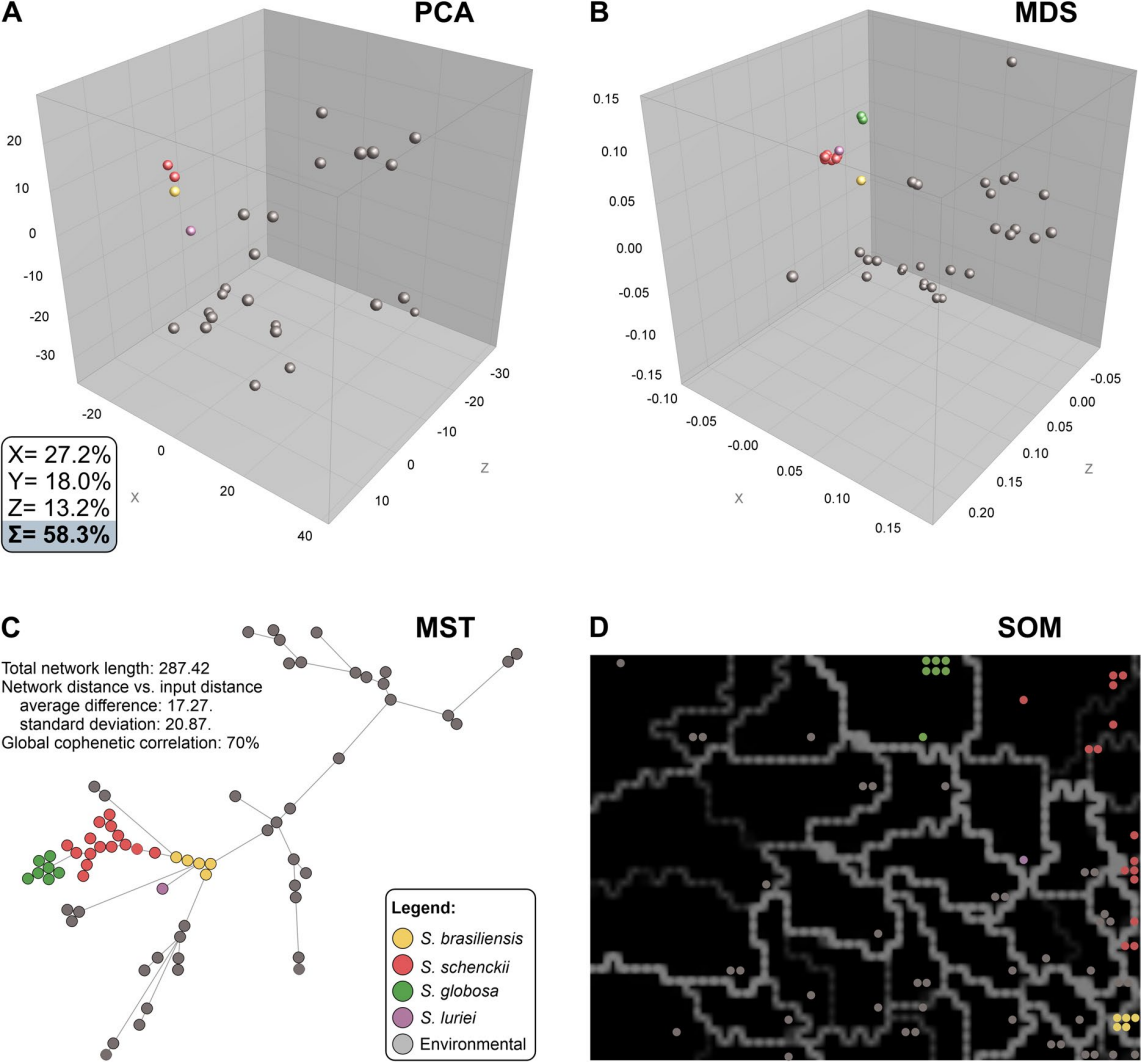
Multivariate population structure and topological analysis of *Sporothrix* genotypes. **A** Principal component analysis (PCA) of genetic diversity; PC1 separates medically relevant *Sporothrix* species from environmental taxa, whereas PC2 discriminates between species within the clinical clade. The first three components explain 58.3% of the total variance. **B** Multidimensional scaling (MDS) plot corroborating the tight clustering of roadkill sequences with clinical reference strains. **C** Minimum spanning tree (MST) revealing the genealogical network topology; short branch lengths indicate high genetic affinity between wildlife isolates and established clinical genotypes (global cophenetic correlation = 70%). **D** Self-organizing map (SOM) analysis projecting roadkill sequences into the same topological neighborhoods as human and veterinary pathogens, which are strictly separated from environmental isolates

To cross-validate the inferred population structure, multivariate statistical approaches, including PCA and MDS, in conjunction with topology-based clustering methods such as the MST and SOM, were applied (Fig. 3). In the PCA (Fig. 3A), the first principal component (PC1) clearly separates the medically relevant *Sporothrix* (*S. brasiliensis*, *S. schenckii*, and *S. globosa*) from the remaining taxa (environmental species), whereas the second principal component (PC2) further discriminates *S. schenckii* and *S. globosa* from *S. brasiliensis*. Together, the first three principal components (PC1–PC3) account for a substantial proportion of the total genetic variance, explaining 58.3% of the observed diversity. This ordination demonstrated that the roadkill-derived sequences clustered tightly with reference clinical isolates, whereas environmental species remained clearly segregated in the multivariate space (Fig. 3B).

Consistent with the PCA and MDS results, the MST analysis revealed a compact and well-resolved network topology (Fig. 3C; model fit statistics detailed in the legend of Fig. 3). Short branch lengths and limited network expansion link wildlife-derived sequences directly to clinically relevant sequences, reinforcing the low genetic divergence among pathogenic genotypes. Reference sequences (environmental species) are shown in gray, providing a contextual framework for the positioning of the newly generated sequences. Finally, the SOM analysis (Fig. 3D) corroborated this distinct partitioning by projecting the roadkill-derived sequences into the same topological neighborhoods as the clinical reference strains, which were strictly separated from the environmental isolates.

Taken together, the congruence among phylogenetic inference (Fig. 2), haplotype networks (Supplementary Table 4), and multivariate clustering (Fig. 3) demonstrated that roadkill samples harbored sequences of *S. brasiliensis*, *S. schenckii*, and *S. globosa*. These findings strongly suggest the involvement of wildlife in the ecoepidemiology of sporotrichosis and highlight the robustness of the analytical framework, even when it is applied to short diagnostic genomic regions.

## Discussion

This study provides novel molecular evidence for the presence of clinical *Sporothrix* spp. in roadkilled wildlife across ecologically diverse, anthropogenically influenced corridors in southeastern Brazil. Historically, the ecoepidemiology of sporotrichosis in Brazil was defined by its high prevalence in domestic animals, predominantly cats (*Felis catus*) [32] and dogs (*Canis lupus familiaris*) [33], with sylvatic reports largely confined to synanthropic rats (*Rattus* spp.) [34], coatis (*Nasua nasua*), capuchin monkeys (*Cebus apella*), and armadillos (*Chlamyphoridae* and *Dasypodidae*) [35]. We expand this established spectrum by detecting *Sporothrix* DNA in taxonomic groups previously unrecognized, such as reptiles, specifically the false coral snake (*Oxyrhopus* spp.); diverse native wild birds, including the picui ground dove (*Columbina picui*), tataupa tinamou (*Crypturellus tataupa*), green-barred woodpecker (*Colaptes melanochloros*), squirrel cuckoo (*Piaya cayana*), and spot-billed toucanet (*Selenidera maculirostris*); and mammals that act as potential bridges for visceral infection, including the southern tiger cat (*Leopardus guttulus*), European hare (*Lepus europaeus*), white-eared opossum (*Didelphis albiventris*), and agouti (*Dasyprocta* spp.).

These findings validate roadkill surveys as a unique window into hidden natural ecological niches, suggesting that sporotrichosis epidemiology extends beyond the classic sapronosis/zoonosis binary, mirroring patterns of other regional zoonoses [36–38]. Our data suggest that the Atlantic Forest acts as a pathogenic incubator under anthropogenic pressure, blurring the boundaries between sylvatic, rural, and urban transmission cycles [39]. The recovery of *Sporothrix* DNA from visceral organs across multiple vertebrates suggests that these animals may act as more than incidental hosts, potentially serving as reservoirs, biological amplifiers, or mechanical vectors [5].

Furthermore, the detection of *Sporothrix* DNA in peri-urban adapted species (*Lepus europaeus*, *Leopardus guttulus*, and *Didelphis albiventris*) points to their possible role as bridge hosts connecting sylvatic and synanthropic cycles [40]. Among these findings, the detection of *S. schenckii* in the heart of the vulnerable southern tiger cat (*L. guttulus*) is particularly noteworthy. Unlike cutaneous lesions typical of feline sporotrichosis [41], cardiac positivity implies hematogenous dissemination, indicating a threat of established systemic mycosis within wild felid populations.

Similarly, the detection of *S. globosa* in *Dasyprocta* spp. (agoutis) challenges the paradigm that this species is solely a low-virulence agent of fixed cutaneous infections [42, 43]. While armadillos (*Dasypus novemcinctus* and *Tolypeutes matacus*) are recognized as carriers owing to their digging behavior [44, 45], agoutis likely encounter inoculum through foraging and seed burial. Visceral detection suggests that under specific conditions, *S. globosa* can achieve dissemination [42]. Furthermore, the diversity of *Sporothrix* in wildlife, including members of the *S. pallida* complex found in other regions, such as *S. davidellisii* and *S. humicola* [18, 46], may represent a reservoir of genetic traits driving the evolution of pathogenicity within *Ophiostomatales* [47].

Our findings regarding *S. brasiliensis* in birds challenge the endothermy-exclusion hypothesis [48] and imply potential for long-distance aerial dispersal [49], complicating containment strategies. Ground-foraging birds, such as *Columbina picui,* may encounter *Sporothrix* propagules in contaminated detritus, particularly in ecotones, considering the high prevalence of this species in open and disturbed environments. Highly mobile birds (*Columbina picui*, *Crypturellus tataupa*, *Colaptes melanochloros*, *Guira guira*, *Selenidera maculirostris*, and *Piaya cayana*) may passively disseminate *Sporothrix* species propagules via avian excreta [49], a mechanism previously documented for mammalian feces [50, 51]. Furthermore, the documented transmission of *S. brasiliensis* from a cockatiel (*Nymphicus hollandicus*) suggests that birds can act as mechanical vectors [52].

The detection of *Sporothrix* in a false coral snake expands the known host spectrum of the pathogen [53]. This finding is consistent with outbreaks in free-ranging pygmy rattlesnakes (*Sistrurus miliarius*), from which *S. schenckii *sensu lato was isolated from skin lesions [53]. Reptiles, often neglected in mycological surveillance, may acquire infection trophically via rodent consumption [54] or through dermal contact, paralleling the ecology of *Ophidiomyces ophiodiicola*, the emerging agent of snake fungal disease [55]. Moreover, reptilian ectothermic thermoregulation might also select for fungal strains with rapid phase transitions, a key virulence factor in thermodimorphic *Sporothrix* species [56].

Current metagenomic evidence suggests that *Sporothrix* ecology is driven by biotic associations [57], whether with nematodes [58], insects [19], or mammals [8], rather than independent soil persistence [59]. This aligns with the “endozoan hypothesis” proposed for *Coccidioides* spp., which posits that the pathogen persists as an endozoan within the host and utilizes the carcass as a privileged, nutrient-rich substrate for saprobic proliferation and sporulation upon host death [60]. Although not statistically significant, the trend toward higher detection during the rainy season aligns with the *Sporothrix* thermohygrophilic profile [61], conditions that likely favor fungal proliferation within these Atlantic Forest microniches. With respect to tissue distribution, the detection of *Sporothrix* DNA predominantly in the heart and liver, rather than the lungs, argues against an inhalational route typical of other systemic mycoses, such as paracoccidioidomycosis [62]. Instead, this visceral pattern supports a mechanism of hematogenous dissemination following traumatic inoculation or trophic acquisition of infected prey or substrate.

## Conclusion

We propose roadkill surveillance as a cost-effective and powerful tool for active monitoring of zoonotic fungal pathogens [10–12]. This strategy provides rapid access to elusive taxa (*e.g.*, wild felids) and functions as an early warning system for environmental pathogen shifts, extending surveillance beyond the domestic cat reservoir [63]. In southeastern Brazil, a region heavily affected by the CTS, the detection of *Sporothrix* DNA across diverse taxa suggests that wildlife are not merely passive components of the ecosystem. While the molecular identification of *S. brasiliensis* in wild birds and *S. schenckii* in the hearts of wild predators highlights critical exposure and potential systemic dissemination, distinguishing between transient DNA carriage, true infection, and active disease will require future complementary histopathological studies.

Nonetheless, this multi-taxa molecular detection signals a significant risk of epidemic spillover into the broader ecosystem [5], underscoring the necessity of a One Health approach that transcends domestic animal control. Anthropogenic drivers, such as deforestation and urbanization, create ecological traps that foster pathogen exchange at forest-agriculture interfaces. Integrating molecular wildlife surveillance into national monitoring programs is therefore essential for predicting and mitigating future sporotrichosis outbreaks.

## Supplementary Information

Below is the link to the electronic supplementary material.

Supplementary file1 (DOCX 62 KB)

## Data Availability

The data presented in this study are available within the article.

## References

[1] Gomes DF, Bueno C, Pinna PH, Woitovicz-Cardoso M, Passos P. March or die: road-killed herpetofauna along BR-040 highway, an ancient road on the Atlantic Forest from Southeastern Brazil. Biota Neotrop. 2023;23(2):e20221454.

[2] Medrano-Vizcaíno P, Espinosa S. Geography of roadkills within the Tropical Andes Biodiversity Hotspot: Poorly known vertebrates are part of the toll. Biotropica. 2021;53(3):820–30. 10.1111/btp.12938.

[3] Coba-Males MA, Medrano-Vizcaíno P, Enríquez S, Brito-Zapata D, Martin-Solano S, Ocaña-Mayorga S, et al. From roads to biobanks: Roadkill animals as a valuable source of genetic data. PLoS ONE. 2023;18(12):e0290836. 10.1371/journal.pone.0290836. 38060478 10.1371/journal.pone.0290836 PMC10703236

[4] Grilo C, Neves T, Bates J, le Roux A, Medrano-Vizcaíno P, Quaranta M, et al. Global Roadkill Data: a dataset on terrestrial vertebrate mortality caused by collision with vehicles. Sci Data. 2025;12(1):505. 10.1038/s41597-024-04207-x. 40164625 10.1038/s41597-024-04207-x PMC11958749

[5] Rodrigues AM, de Hoog GS, de Camargo ZP. *Sporothrix* species causing outbreaks in animals and humans driven by animal-animal transmission. PLoS Pathog. 2016;12(7):e1005638. 10.1371/journal.ppat.1005638. 27415796 10.1371/journal.ppat.1005638 PMC4945023

[6] de Carvalho JA, Beale MA, Hagen F, Fisher MC, Kano R, Bonifaz A, et al. Trends in the molecular epidemiology and population genetics of emerging *Sporothrix* species. Stud Mycol. 2021;100:100129. 10.1016/j.simyco.2021.100129. 35027980 10.1016/j.simyco.2021.100129 PMC8693333

[7] Gremião ID, da Rocha EMS, Montenegro H, Carneiro AJB, Xavier MO, de Farias MR, et al. Guideline for the management of feline sporotrichosis caused by *Sporothrix brasiliensis* and literature revision. Braz J Microbiol. 2021;52(1):107–24. 10.1007/s42770-020-00365-3. 32990922 10.1007/s42770-020-00365-3 PMC7966609

[8] Rodrigues AM, Della Terra PP, Gremiao ID, Pereira SA, Orofino-Costa R, de Camargo ZP. The threat of emerging and re-emerging pathogenic *Sporothrix* species. Mycopathologia. 2020;185(5):813–42. 10.1007/s11046-020-00425-0. 32052359 10.1007/s11046-020-00425-0

[9] Della Terra PP, Gonsales FF, de Carvalho JA, Hagen F, Kano R, Bonifaz A, et al. Development and evaluation of a multiplex qPCR assay for rapid diagnostics of emerging sporotrichosis. Transbound Emerg Dis. 2022;69(4):e704–16. 10.1111/tbed.14350. 34687495 10.1111/tbed.14350

[10] Monsalve-Lara J, Drummond M, Romero-Alvarez D, Velho P, Jiménez-García D, Marques R, et al. Prevalence of *Mycobacterium leprae* and *Mycobacterium lepromatosis* in roadkill armadillos in Brazil. Acta Trop. 2024;258:107333. 10.1016/j.actatropica.2024.107333. 39067841 10.1016/j.actatropica.2024.107333

[11] Duarte MD, Henriques AM, Barros SC, Fagulha T, Mendonça P, Carvalho P, et al. Snapshot of viral infections in wild carnivores reveals ubiquity of parvovirus and susceptibility of Egyptian mongoose to feline panleukopenia virus. PLoS ONE. 2013;8(3):e59399. 10.1371/journal.pone.0059399. 23527182 10.1371/journal.pone.0059399 PMC3603882

[12] Richini-Pereira VB, Marson PM, Hayasaka EY, Victoria C, da Silva RC, Langoni H. Molecular detection of **Leishmani*a* spp. in road-killed wild mammals in the Central Western area of the State of São Paulo, Brazil. J Venom Anim Toxins Incl Trop Dis. 2014;20:27. 10.1186/1678-9199-20-27. 24963288 10.1186/1678-9199-20-27 PMC4068874

[13] Silva MA, Guideli LC, Neto AC, de Brum PL, Kormann ACM. Multiple correspondence analysis applied to the study of the relationship between traffic crashes and precipitation on a highway in Brazil. Transportes. 2020;28(3):196–211.

[14] Pereira AD, Yabu MHS, Geller IV, Lehn CR, Vidotto-Magnoni AP, Bogoni JA, et al. Don’t speed up, speed kills: Mammal roadkills on highway sections of PR-445 in the South of Brazil. Oecologia Australis. 2021;25(1):34–46.

[15] Caldart ET, Sevá AdP, Pinto-Ferreira F, Pereira Pachoal AT, de Oliveira JS, Cortela IdB, et al. American cutaneous leishmaniasis associated with degradation of native forest, regardless of economic, social and infrastructure vulnerability. Zoonoses Public Health. 2021;68(4):327–43. 10.1111/zph.12793. 33340442 10.1111/zph.12793

[16] Kitano T, Umetsu K, Tian W, Osawa M. Two universal primer sets for species identification among vertebrates. Int J Legal Med. 2007;121(5):423–7. 10.1007/s00414-006-0113-y. 16845543 10.1007/s00414-006-0113-y

[17] Hall TA. BioEdit: a user-friendly biological sequence alignment editor and analysis program for Windows 95/98/NT. Nucleic Acids Symp Ser. 1999;41:95–8.

[18] Kidd SE, Sandoval-Denis M, Malik R, Hagen F, Rodrigues AM. *Sporothrix davidellisii*: a new pathogenic species belonging to the *Sporothrix pallida* complex. Med Mycol. 2025;63(4):myaf034. 10.1093/mmy/myaf034. 40216404 10.1093/mmy/myaf034 PMC12015470

[19] de Beer ZW, Procter M, Wingfield MJ, Marincowitz S, Duong TA. Generic boundaries in the Ophiostomatales reconsidered and revised. Stud Mycol. 2022;101:57–120. 10.3114/sim.2022.101.02. 36059894 10.3114/sim.2022.101.02 PMC9365045

[20] Rodrigues AM, Cruz Choappa R, Fernandes GF, De Hoog GS, Camargo ZP. *Sporothrix chilensis* sp. nov. (Ascomycota: Ophiostomatales), a soil-borne agent of human sporotrichosis with mild-pathogenic potential to mammals. Fungal Biol. 2016;120(2):246–64. 10.1016/j.funbio.2015.05.006. 26781380 10.1016/j.funbio.2015.05.006

[21] de Beer ZW, Duong TA, Wingfield MJ. The divorce of *Sporothrix* and *Ophiostoma*: solution to a problematic relationship. Stud Mycol. 2016;83:165–91. 10.1016/j.simyco.2016.07.001. 27616802 10.1016/j.simyco.2016.07.001 PMC5007658

[22] Sneath PH, Sokal RR. Unweighted pair group method with arithmetic mean. Numer Taxon. 1973; 230–4.

[23] Felsenstein J. Evolution confidence limits on phylogenies: An approach using the bootstrap. Evolution. 1985;39(4):783–91. 28561359 10.1111/j.1558-5646.1985.tb00420.x

[24] Tamura K, Stecher G, Kumar S. MEGA11: Molecular Evolutionary Genetics Analysis Version 11. Mol Biol Evol. 2021;38(7):3022–7. 10.1093/molbev/msab120. 33892491 10.1093/molbev/msab120 PMC8233496

[25] Novembre J, Stephens M. Interpreting principal component analyses of spatial population genetic variation. Nat Genet. 2008;40(5):646–9. 10.1038/ng.139. 18425127 10.1038/ng.139 PMC3989108

[26] Prim RC. Shortest connection networks and some generalizations. Bell Syst Tech J. 1957;36(6):1389–401. 10.1002/j.1538-7305.1957.tb01515.x.

[27] Kohonen T. Self-Organizing Maps. 3 ed 2001.

[28] Vesanto J, Himberg J, Alhoniemi E, Parhankangas J, editors. Self-organizing map in Matlab: the SOM Toolbox. Proceedings of the Matlab DSP conference. Espoo, Finland: Matlab DSP Conference; 1999

[29] Rodrigues AM, Beale MA, Hagen F, Fisher MC, Terra PPD, de Hoog S, et al. The global epidemiology of emerging *Histoplasma* species in recent years. Stud Mycol. 2020;97:100095. 10.1016/j.simyco.2020.02.001. 33335607 10.1016/j.simyco.2020.02.001 PMC7714791

[30] Rozas J, Ferrer-Mata A, Sanchez-DelBarrio JC, Guirao-Rico S, Librado P, Ramos-Onsins SE, et al. DnaSP 6: DNA sequence polymorphism analysis of large data sets. Mol Biol Evol. 2017;34(12):3299–302. 10.1093/molbev/msx248. 29029172 10.1093/molbev/msx248

[31] Bandelt HJ, Forster P, Röhl A. Median-joining networks for inferring intraspecific phylogenies. Mol Biol Evol. 1999;16(1):37–48. 10331250 10.1093/oxfordjournals.molbev.a026036

[32] Gremião ID, Miranda LH, Reis EG, Rodrigues AM, Pereira SA. Zoonotic epidemic of sporotrichosis: cat to human transmission. PLoS Pathog. 2017;13(1):e1006077. 10.1371/journal.ppat.1006077. 28103311 10.1371/journal.ppat.1006077 PMC5245785

[33] Baes Pereira S, dos Reis Gomes A, Bressan Waller S, Batista Xavier JR, Messias Rodrigues A, Kutscher Ripoll M, et al. Sporotrichosis in dogs: epidemiological and clinical-therapeutic profile and the emergence of itraconazole-resistant isolates. Med Mycol. 2022;60(12):myac089. 10.1093/mmy/myac089. 36455616 10.1093/mmy/myac089

[34] Lutz A, Splendore A. [On a mycosis observed in men and mice: contribution to the knowledge of the so-called sporotrichosis]. Rev Med Sao Paulo. 1907;21:443–50.

[35] Rabello VBS, Almeida MA, Bernardes-Engemann AR, Almeida-Paes R, de Macedo PM, Zancopé-Oliveira RM. The historical burden of sporotrichosis in Brazil: a systematic review of cases reported from 1907 to 2020. Braz J Microbiol. 2022;53(1):231–44. 10.1007/s42770-021-00658-1. 34825345 10.1007/s42770-021-00658-1 PMC8882507

[36] Vieira AS, D’Andrea PS, Vilela RdV, Loretto D, Jaeger LH, Carvalho-Costa FA, et al. Pathogenic *Leptospira* species are widely disseminated among small mammals in Atlantic Forest biome. Transbound Emerg Dis. 2019;66(3):1195–201. 10.1111/tbed.13135. 30703279 10.1111/tbed.13135

[37] Muylaert RL, Bovendorp RS, Sabino-Santos G Jr., Prist PR, Melo GL, Priante CdF, et al. Hantavirus host assemblages and human disease in the Atlantic Forest. PLoS Negl Trop Dis. 2019;13(8):e0007655. 10.1371/journal.pntd.0007655. 31404077 10.1371/journal.pntd.0007655 PMC6748440

[38] Rozental T, Ferreira MS, Guterres A, Mares-Guia MA, Teixeira BR, Gonçalves J, et al. Zoonotic pathogens in Atlantic Forest wild rodents in Brazil: *Bartonella* and *Coxiella* infections. Acta Trop. 2017;168:64–73. 10.1016/j.actatropica.2017.01.003. 28077317 10.1016/j.actatropica.2017.01.003

[39] Palmeirim AF, Barreto JR, Prist PR. The importance of Indigenous Lands and landscape structure in shaping the zoonotic disease risk—insights from the Brazilian Atlantic Forest. One Health. 2025;21:101104. 10.1016/j.onehlt.2025.101104. 40599645 10.1016/j.onehlt.2025.101104 PMC12209932

[40] Morens DM, Folkers GK, Fauci AS. The challenge of emerging and re-emerging infectious diseases. Nature. 2004;430(6996):242–9. 10.1038/nature02759. 15241422 10.1038/nature02759 PMC7094993

[41] Gremiao ID, Menezes RC, Schubach TM, Figueiredo AB, Cavalcanti MC, Pereira SA. Feline sporotrichosis: epidemiological and clinical aspects. Med Mycol. 2015;53(1):15–21. 10.1093/mmy/myu061. 25477076 10.1093/mmy/myu061

[42] Arrillaga-Moncrieff I, Capilla J, Mayayo E, Marimon R, Mariné M, Gené J, et al. Different virulence levels of the species of *Sporothrix* in a murine model. Clin Microbiol Infect. 2009;15(7):651–5. 10.1111/j.1469-0691.2009.02824.x. 19624508 10.1111/j.1469-0691.2009.02824.x

[43] Fernandes GF, dos Santos PO, Rodrigues AM, Sasaki AA, Burger E, de Camargo ZP. Characterization of virulence profile, protein secretion and immunogenicity of different *Sporothrix schenckii sensu stricto* isolates compared with *S. globosa* and *S. brasiliensis* species. Virulence. 2013;4(3):241–9. 10.4161/viru.23112. 23324498 10.4161/viru.23112 PMC3711982

[44] Rodrigues AM, de Carvalho JA, Nery AF, Hueb M, Garcia I, Guevara A, et al. Multifocal sporotrichosis associated with armadillo hunting in midwest Brazil: An in-depth case study and comprehensive literature analysis. Mycopathologia. 2024;189(4):53. 10.1007/s11046-024-00854-1. 38864961 10.1007/s11046-024-00854-1

[45] Rodrigues AM, Bagagli E, de Camargo ZP, Bosco SM. *Sporothrix**schenckii**sensu**stricto* isolated from soil in an armadillo’s burrow. Mycopathologia. 2014;177(3–4):199–206. 10.1007/s11046-014-9734-8. 24577793 10.1007/s11046-014-9734-8

[46] Nesseler A, Schauerte N, Geiger C, Kaerger K, Walther G, Kurzai O, et al. *Sporothrix**humicola* (Ascomycota: *Ophiostomatales*) - A soil-borne fungus with pathogenic potential in the eastern quoll (*Dasyurus**viverrinus*). Med Mycol Case Rep. 2019;25:39–44. 10.1016/j.mmcr.2019.07.008. 31428554 10.1016/j.mmcr.2019.07.008 PMC6695275

[47] Naliato GFS, Arantes TD, Rodrigues AM, Pimentel de Barros P, Castanho Scortecci K, Fernanda França G, et al. Virulence assessment of *Sporothrix* species from clinical and environmental clades using *Tenebrio molitor* as an experimental model. Med Mycol. 2025. 10.1093/mmy/myaf092. 41074848 10.1093/mmy/myaf092

[48] Robert VA, Casadevall A. Vertebrate endothermy restricts most fungi as potential pathogens. J Infect Dis. 2009;200(10):1623–6. 10.1086/644642. 19827944 10.1086/644642

[49] Johansson NR, Kaasalainen U, Rikkinen J. Diversity of fungi attached to birds corresponds to the habitat ecologies of their avian dispersal vectors. Ann Bot. 2025. 10.1093/aob/mcaf077. 40318186 10.1093/aob/mcaf077 PMC12464955

[50] Rodrigues AM, de Hoog GS, de Camargo ZP. Molecular diagnosis of pathogenic Sporothrix species. PLoS Negl Trop Dis. 2015;9(12):e0004190. 10.1371/journal.pntd.0004190. 26623643 10.1371/journal.pntd.0004190 PMC4666615

[51] Montenegro H, Rodrigues AM, Galvão Dias MA, da Silva EA, Bernardi F, Camargo ZP. Feline sporotrichosis due to *Sporothrix**brasiliensis*: an emerging animal infection in São Paulo, Brazil. BMC Vet Res. 2014;10(1):269. 10.1186/s12917-014-0269-5. 25407096 10.1186/s12917-014-0269-5 PMC4244058

[52] Fichman V, Gremião IDF, Mendes-Júnior AAV, Sampaio FMS, Freitas DFS, Oliveira MME, et al. Sporotrichosis transmitted by a cockatiel (*Nymphicus**hollandicus*). J Eur Acad Dermatol Venereol. 2018;32(4):e157–8. 10.1111/jdv.14661. 29080316 10.1111/jdv.14661

[53] Cheatwood JL, Jacobson ER, May PG, Farrell TM, Homer BL, Samuelson DA, et al. An outbreak of fungal dermatitis and stomatitis in a free-ranging population of pigmy rattlesnakes (**Sistrurus miliarius barbour*i*) in Florida. J Wildl Dis. 2003;39(2):329–37. 10.7589/0090-3558-39.2.329. 12910760 10.7589/0090-3558-39.2.329

[54] Schilliger L, Paillusseau C, François C, Bonwitt J. Major emerging fungal diseases of reptiles and amphibians. Pathogens. 2023. 10.3390/pathogens12030429. 36986351 10.3390/pathogens12030429 PMC10053826

[55] Lorch JM, Lankton J, Werner K, Falendysz EA, McCurley K, Blehert DS. Experimental infection of snakes with *Ophidiomyces ophiodiicola* causes pathological changes that typify snake fungal disease. MBio. 2015;6(6):e01534-15. 10.1128/mBio.01534-15. 26578676 10.1128/mBio.01534-15 PMC4659463

[56] García-Carnero LC, Martínez-Álvarez JA. Virulence factors of *Sporothrix**schenckii*. J Fungi. 2022. 10.3390/jof8030318. 10.3390/jof8030318 PMC8949611 35330320

[57] Andersen B, Frisvad JC, Søndergaard I, Rasmussen IS, Larsen LS. Associations between fungal species and water-damaged building materials. Appl Environ Microbiol. 2011;77(12):4180–8. 10.1128/AEM.02513-10. 21531835 10.1128/AEM.02513-10 PMC3131638

[58] Jumbam B, Amiri Z-B, Dandurand L-M, Zasada IA, Aime MC. Analyses of fungal communities from culture-dependent and -independent studies reveal novel mycobiomes associated with *Globodera* and *Heterodera* species. Phytobiomes J. 2024;8(4):621–42. 10.1094/PBIOMES-11-23-0122-R.

[59] Criseo G, Romeo O. Ribosomal DNA sequencing and phylogenetic analysis of environmental **Sporothrix schencki*i* strains: comparison with clinical isolates. Mycopathologia. 2010;169(5):351–8. 10.1007/s11046-010-9274-9. 20119849 10.1007/s11046-010-9274-9

[60] Taylor JW, Barker BM. The endozoan, small-mammal reservoir hypothesis and the life cycle of *Coccidioides* species. Med Mycol. 2019;57(Supplement_1):S16–20. 10.1093/mmy/myy039. 30690603 10.1093/mmy/myy039 PMC6702415

[61] Ramírez-Soto MC, Aguilar-Ancori E, Tirado-Sánchez A, Bonifaz A. Ecological determinants of sporotrichosis etiological agents. J Fungi. 2018;4(3):95. 10.3390/jof4030095 PMC6162718 30103554

[62] Rodrigues AM, Hagen F, Puccia R, Hahn RC, de Camargo ZP. *Paracoccidioides* and paracoccidioidomycosis in the 21st century. Mycopathologia. 2023;188(1–2):129–33. 10.1007/s11046-022-00704-y. 36633737 10.1007/s11046-022-00704-y

[63] Almeida-Silva F, Rabello VB, Scramignon-Costa BD, Zancopé-Oliveira RM, de Macedo PM, Almeida-Paes R. Beyond domestic cats: Environmental detection of *Sporothrix**brasiliensis* DNA in a hyperendemic area of sporotrichosis in Rio de Janeiro state. Brazil J Fungi. 2022. 10.3390/jof8060604. 10.3390/jof8060604 PMC9224889 35736087

