# Supplementary material for: Molecular detection of medically relevant Sporothrix species in roadkilled wildlife in the Brazilian Atlantic forest
Source: Mycopathologia. 2026 Mar 12;191(2):42. doi: 10.1007/s11046-026-01067-4 (PMC12979292; doi:10.1007/s11046-026-01067-4)
Supplement: Supplementary file 1 — Supplementary file1 (DOCX 62 KB) [file 11046_2026_1067_MOESM1_ESM.docx]

**Supplementary Table 1.** Detection of *Sporothrix* spp. DNA in road-killed wildlife from Paraná, Brazil (2017–2023).

| **Scientific name** | **Tissue** | **DNA [ng/µl]** | **DNA 260/280** | **Collection period** | **Latitude** | **Longitude** | **Sex** | **Development stage** |
| --- | --- | --- | --- | --- | --- | --- | --- | --- |
| *Salvator merianae* | Liver | 14.7 | 1.93 | Spring/2017 | 23°19'13"S | 51°12'34"W | Male | Juvenile |
| *Salvator merianae* | Heart | 829.0 | 1.92 | Summer/2017 | 23°19'2"S | 51°11'3"W | Female | Juvenile |
| *Leopardus guttulus* | Heart | 52.4 | 2.06 | Summer/2017 | 23°36'33"S | 51°10'54"W | Female | Adult |
| *Didelphis albiventris* | Heart | 105.1 | 1.83 | Winter/2017 | 23°20'50"S | 51°11'43"W | Female | Juvenile |
| *Lepus europaeus* | Heart | 138.1 | 1.97 | Summer/2017 | 23°18'12"S | 51°10'15"W | Male | Adult |
|  | Liver | 147.0 | 1.94 |  |  |  |  |  |
|  | Lung | 575.5 | 1.89 |  |  |  |  |  |
| *Tamandua tetradactyla* | Heart | 470.8 | 1.90 | Autumn/2017 | 23°16'9"S | 51°16'37"W | Male | Adult |
|  | Liver | 175.2 | 1.68 |  |  |  |  |  |
|  | Lung | 1051.2 | 1.94 |  |  |  |  |  |
| *Leopardus guttulus* | Heart | 284.6 | 1.86 | Autumn/2017 | 23°56'24"S | 51°17'26"W | Female | Juvenile |
| *Columbina picui* | Heart | 254.6 | 1.95 | Spring/2017 | 23°43'3"S | 51°23'47"W | Male | N/A |
|  | Liver | 191.1 | 1.87 |  |  |  |  |  |
|  | Lung | 965.8 | 1.95 |  |  |  |  |  |
| *Procyon cancrivorus* | Heart | 275.5 | 2.01 | Spring/2017 | 23°54'28"S | 51°11'35"W | Male | Adult |
| *Athene cunicularius* | Heart | 1599.3 | 1.89 | Spring/2017 | 23°11'46"S | 51°12'43"W | Male | Adult |
| *Vanellus chilensis* | Heart | 392.0 | 2.00 | Spring/2017 | 23°15'2"S | 51°13'21"W | Male | Juvenile |
|  | Liver | 124.8 | 2.05 |  |  |  |  |  |
| *Columbina minuta* | Heart | 440.9 | 1.93 | Spring/2017 | 23°15'2"S | 51°13'21"W | N/A | Adult |
|  | Liver | 824.2 | 1.92 |  |  |  |  |  |
| *Coragyps atratus* | Heart | 251.0 | 1.93 | Winter/2017 | 23°18'45"S | 51°9'33"W | Male | Adult |
|  | Liver | 712.1 | 1.91 |  |  |  |  |  |
|  | Lung | 758.2 | 1.89 |  |  |  |  |  |
| *Puma concolor* | Liver | 69.5 | 1.71 | Autumn/2017 | 23°1'52"S | 50°48'29"W | Female | Juvenile |
| *Didelphis albiventris* | Heart | 194.5 | 1.97 | Spring/2017 | 23°36'33"S | 51°10'54"W | Male | Juvenile |
|  | Lung | 1946.9 | 1.95 |  |  |  |  |  |
| *Crypturellus tataupa* | Heart | 484.5 | 1.95 | Winter/2017 | 23°30'38"S | 51°14'3"W | Female | N/A |
| *Patagioenas picazuro* | Liver | 585.2 | 1.92 | Summer/2017 | 23°19'20"S | 51°11'38"W | Male | Adult |
| *Didelphis albiventris* | Lung | 607.0 | 1.94 | Winter/2017 | 23°18'55"S | 51°8'6"W | Male | Adult |
| *Salvator merianae* | Heart | 250.0 | 1.97 | Spring/2017 | 23°19'54"S | 51°9'45"W | Female | Adult |
|  | Liver | 29.6 | 1.95 |  |  |  |  |  |
|  | Lung | 889.5 | 1.93 |  |  |  |  |  |
| *Tamandua tetradactyla* | Heart | 219.7 | 1.93 | Spring/2017 | 23°39'38"S | 52°34'2"W | Male | Adult |
|  | Liver | 24.7 | 1.82 |  |  |  |  |  |
|  | Lung | 204.2 | 2.03 |  |  |  |  |  |
| *Dasypus novemcinctus* | Heart | 193.9 | 1.92 | Spring/2017 | 23°19'3"S | 51°12'30"W | Male | Adult |
|  | Liver | 144.7 | 2.04 |  |  |  |  |  |
|  | Lung | 168.9 | 1.89 |  |  |  |  |  |
| *Colaptes melanochloros* | Heart | 270.8 | 1.89 | Spring/2017 | 23°36'57"S | 51°18'36"W | NS | Juvenile |
|  | Liver | 1154.2 | 1.89 |  |  |  |  |  |
|  | Lung | 863.6 | 1.89 |  |  |  |  |  |
| *Coragyps atratus* | Heart | 587.1 | 1.90 | Spring/2017 | 23°11'30"S | 50°47'4"W | Male | Adult |
|  | Liver | 147.6 | 1.90 |  |  |  |  |  |
|  | Lung | 319.1 | 1.88 |  |  |  |  |  |
|  | Spleen | 2016.5 | 1.91 |  |  |  |  |  |
| *Guira guira* | Heart | 493.6 | 1.94 | Spring/2017 | 23°3'43"S | 50°54'27"W | Male | Juvenile |
|  | Liver | 105.2 | 1.96 |  |  |  |  |  |
|  | Lung | 974.4 | 1.93 |  |  |  |  |  |
| *Tamandua tetradactyla* | Heart | 4.1 | 2.37 | Spring/2017 | 23°56'11"S | 51°33'57"W | Female | Adult |
|  | Liver | 13.8 | 2.20 |  |  |  |  |  |
|  | Lung | 26.2 | 2.14 |  |  |  |  |  |
| *Athene cunicularia* | Liver | 1333.2 | 1.89 | Spring/2017 | 23°30'1"S | 51°45'41"W | Female | Juvenile |
| *Caracara plancus* | Heart | 281.6 | 1.92 | Spring/2017 | 23°28'48"S | 51°59'59"W | Male | Juvenile |
|  | Heart | 145.6 | 1.93 |  |  |  |  |  |
| *Pitangus sulphuratus* | Liver | 24.4 | 2.11 | Winter/2017 | 23°19'25"S | 51°11'59"W | Female | Offspring |
| *Tangara sayaca* | Heart | 300.1 | 1.88 | Autumn/2017 | N/A | N/A | N/A | Offspring |
|  | Liver | 458.7 | 1.94 |  |  |  |  |  |
| *Megascops choliba* | Liver | 260.6 | 1.91 | Autumn/2017 | 23°19'75"S | 51°12'11"W | Male | Offspring |
| *Selenidera maculirostris* | Liver | 226.0 | 1.90 | Summer/2018 | 23°14'46"S | 51°56'22"W | Male | Juvenile |
| *Coendou spinosus* | Heart | 271.5 | 1.88 | Winter/2018 | 23°42'54.0"S | 50°43'36.7"W | N/A | N/A |
|  | Liver | 581.6 | 1.93 |  |  |  |  |  |
|  | Lung | 572.4 | 1.90 |  |  |  |  |  |
| *Sapajus nigritus* | Heart | 134.1 | 1.95 | Winter/2018 | 23°35'18"S | 51°36'45.6"W | N/A | N/A |
|  | Liver | 1996.0 | 1.97 |  |  |  |  |  |
|  | Lung | 22.9 | 1.98 |  |  |  |  |  |
| *Rhynchotus rufescens* | Heart | 153.1 | 1.99 | Winter/2018 | 23°15'00"S | 50°57'72"W | Female | Adult |
|  | Liver | 5230.1 | 2.17 |  |  |  |  |  |
| *Cerdocyon thous* | Heart | 257.5 | 2.01 | Winter/2020 | 23°26'17''S | 51°8'19''W | Male | N/A |
|  | Lung | 653.0 | 1.97 |  |  |  |  |  |
| *Numida meleagris* | Lung | 516.3 | 1.93 | Winter/2020 | 23°28'16.8''S | 51°8'4.08''W | N/A | N/A |
| *Patagioenas picazuro* | Lung | 158.1 | 1.84 | Winter/2020 | 23°27'11''S | 51°7'51''W | N/A | Juvenile |
| *Leopardus guttulus* | Heart | 238.8 | 1.71 | Summer/2020 | 23°28'47'31''S | 51°7'57.816''W | Male | Juvenile |
|  | Lung | 2713.2 | 1.95 |  |  |  |  |  |
| *Megascops choliba* | Liver | 3327.3 | 2.01 | Summer/2020 | 23°27'9.81''S | 51°7'51.198''W | Female | Juvenile |
|  | Lung | 3434.7 | 1.92 |  |  |  |  |  |
| *Columba livia* | Heart | 190.3 | 1.94 | Summer/2020 | 23°28'33.76''S | 51°7'57.762''W | Female | Adult |
|  | Liver | 514.7 | 2.02 |  |  |  |  |  |
|  | Lung | 478.2 | 1.92 |  |  |  |  |  |
| *Oxyrhopus* spp. | Liver | 402.4 | 2.11 | Winter/2020 | N/A | N/A | Female | Adult |
| *Penelope superciliaris* | Lung | 297.7 | 1.92 | Spring/2020 | 23°28'54.906''S | 51°7'58.83''W | Female | N/A |
| *Myiodynastes maculatus* | Lung | 1283.6 | 1.92 | Autumn/2020 | 23°25'10.446''S | 51°8'21.57''W | N/A | Juvenile |
| *Piaya cayana* | Liver | 993.5 | 2.10 | Autumn/2020 | 23°28'0.216''S | 51°8'2.202''W | N/A | N/A |
|  | Lung | 3737.9 | 1.92 |  |  |  |  |  |
| *Guira guira* | Lung | 635.1 | 1.96 | Spring/2020 | 23°28'46.362''S | 51°7'56.808''W | Female | Adult |
| *Patagioenas picazuro* | Heart | 177.5 | 1.73 | Spring/2020 | 23°27'6.684''S | 51°7'52.494''W | Female | Adult |
|  | Liver | 2230.4 | 2.04 |  |  |  |  |  |
|  | Lung | 3141.2 | 1.87 |  |  |  |  |  |
| *Tyto furcata* | Heart | 484.1 | 1.92 | Summer/2020 | 23°28'16.8''S | 51°8'4.08''W | Male | N/A |
|  | Lung | 663.2 | 1.93 |  |  |  |  |  |
| *Columbina talpacota* | Lung | 316.6 | 1.92 | Winter/2021 | 23°26'55.56''S | 51°7'56.052''W | Male | N/A |
| *Crotophaga ani* | Lung | 2527.5 | 2.01 | Spring/2021 | 23°25'6.12''S | 51°8'19.098''W | Male | N/A |
| *Megascops choliba* | Lung | 450.6 | 1.84 | Winter/2021 | 23°19'42.2"S | 51°11'21.4"W | Female | N/A |
| *Didelphis albiventris* | Lung | 1526.1 | 1.88 | Winter/2021 | 23°19'37.2"S | 51°12'14.4"W | N/A | N/A |
|  | Spleen | 3284.9 | 1.92 |  |  |  |  |  |
| *Guira guira* | Liver | 353.5 | 2.18 | Winter/2021 | 23°19'42.234''S | 51°11'21.4''W | Male | N/A |
|  | Lung | 2602.8 | 1.90 |  |  |  |  |  |
| *Coendou spinosus* | Liver | 727.3 | 2.06 | Spring/2022 | 23°19'35.7"S | 51°11'25.1"W | Male | Adult |
|  | Lung | 234.5 | 1.89 |  |  |  |  |  |
| *Lepus europaeus* | Heart | 5.7 | 2.54 | Spring/2022 | 23°18'49.9"S | 51°12'53.4"W | Male | Adult |
|  | Liver | 16.6 | 2.85 |  |  |  |  |  |
|  | Lung | 10.2 | 2.69 |  |  |  |  |  |
| *Didelphis albiventris* | Heart | 502.6 | 1.94 | Autumn/2022 | 23°54'14.1"S | 51°11'26.1"W | Male | Juvenile |
|  | Liver | 713.1 | 1.97 |  |  |  |  |  |
|  | Lung | 3123.2 | 1.98 |  |  |  |  |  |
|  | Spleen | 731.9 | 1.96 |  |  |  |  |  |
| *Didelphis albiventris* | Liver | 282.0 | 1.88 | Winter/2022 | 23°19'19.0"S | 51°12'00.1"W | Female | Juvenile |
|  | Lung | 1758.8 | 1.93 |  |  |  |  |  |
|  | Spleen | 2305.7 | 1.96 |  |  |  |  |  |
| *Boa constrictor* | Liver | 267.4 | 2.08 | Winter/2022 | N/A | N/A | Female | Adult |
|  | Lung | 139.6 | 1.99 |  |  |  |  |  |
| *Didelphis albiventris* | Liver | 702.1 | 1.94 | Summer/2022 | 23°21'42.4"S | 51°11'56.2"W | Female | Adult |
|  | Lung | 978.5 | 1.90 |  |  |  |  |  |
| *Didelphis albiventris* | Heart | 154.5 | 2.01 | Winter/2022 | N/A | N/A | Female | Adult |
|  | Lung | 1057.6 | 1.96 |  |  |  |  |  |
|  | Spleen | 2170.5 | 1.96 |  |  |  |  |  |
| *Sapajus nigritus* | Heart | 228.5 | 1.89 | Winter/2022 | N/A | N/A | Male | Adult |
|  | Liver | 160.4 | 1.67 |  |  |  |  |  |
|  | Lung | 866.0 | 1.90 |  |  |  |  |  |
|  | Spleen | 3378.4 | 1.88 |  |  |  |  |  |
| *Didelphis albiventris* | Heart | 203.9 | 1.89 | Winter/2022 | 23°17'26.4"S | 51°16'43.4"W | Female | Adult |
|  | Liver | 786.6 | 1.95 |  |  |  |  |  |
|  | Lung | 352.8 | 1.95 |  |  |  |  |  |
|  | Spleen | 3926.0 | 1.88 |  |  |  |  |  |
| *Guira guira* | Lung | 798.5 | 1.94 | Spring/2022 | 23°19'40.7"S | 51°11'36.1"W | Male | Adult |
| *Guira guira* | Liver | 53.5 | 1.99 | Spring/2022 | 23°19'40.7"S | 51°11'36.1"W | Female | Adult |
|  | Lung | 845.5 | 1.94 |  |  |  |  |  |
| *Patagioenas picazuro* | Heart | 336.4 | 1.91 | Spring/2022 | 23°19'36.5"S | 51°09'24.1"W | Female | Adult |
|  | Liver | 1258.2 | 1.93 |  |  |  |  |  |
| *Dasypus novemcinctus* | Heart | 162.7 | 1.95 | Spring/2022 | 23°20'48.2"S | 51°10'26.4"W | Male | Adult |
|  | Liver | 443.0 | 1.85 |  |  |  |  |  |
|  | Lung | 1932.2 | 1.94 |  |  |  |  |  |
| *Nasua nasua* | Heart | 181.8 | 1.89 | Spring/2022 | 23°18'24.9"S | 51°10'49.5"W | Male | Adult |
|  | Liver | 216.9 | 1.69 |  |  |  |  |  |
|  | Lung | 413.9 | 1.90 |  |  |  |  |  |
|  | Spleen | 2172.5 | 1.92 |  |  |  |  |  |
| *Didelphis albiventris* | Lung | 2390.9 | 1.92 | Spring/2023 | 23°19'02.9"S | 51°11'49.0"W | Male | Adult |
|  | Spleen | 13.6 | 2.06 |  |  |  |  |  |
| *Didelphis albiventris* | Heart | 731.0 | 2.02 | Spring/2023 | N/A | N/A | Female | Adult |
|  | Lung | 400.4 | 1.98 |  |  |  |  |  |
|  | Spleen | 3434.1 | 1.93 |  |  |  |  |  |
| *Hydrochoerus hydrochaeris* | Heart | 85.6 | 2.04 | Summer/2023 | 23°20'31.3"S | 51°08'09.9"W | Female | Adult |
|  | Lung | 235.0 | 1.99 |  |  |  |  |  |
| *Didelphis albiventris* | Heart | 140.9 | 2.03 | Autumn/2023 | 23°19'32.1"S | 51°11'51.5"W | Female | Adult |
|  | Liver | 271.2 | 2.02 |  |  |  |  |  |
|  | Lung | 488.0 | 1.94 |  |  |  |  |  |
|  | Spleen | 1947.7 | 1.96 |  |  |  |  |  |
| *Crotalus durissus* | Heart | 197.3 | 2.06 | Autumn/2023 | N/A | N/A | Male | Adult |
|  | Liver | 1014.4 | 2.12 |  |  |  |  |  |
|  | Lung | 333.0 | 2.16 |  |  |  |  |  |
| *Didelphis albiventris* | Heart | 1020.5 | 1.94 | Autumn/2023 | 23°19'14.5"S | 51°11'38.4"W | Male | Adult |
|  | Liver | 15.8 | 1.85 |  |  |  |  |  |
|  | Lung | 175.1 | 1.95 |  |  |  |  |  |
| *Dasyprocta azarae* | Heart | 198.6 | 1.97 | Autumn/2023 | 23°20'47.0"S | 51°08'32.9"W | Male | Adult |
|  | Liver | 3117.4 | 2.07 |  |  |  |  |  |
|  | Lung | 593.2 | 1.97 |  |  |  |  |  |
|  | Spleen | 4422.8 | 1.84 |  |  |  |  |  |
| *Cerdocyon thous* | Liver | 2541.4 | 2.05 | Winter/2023 | 23°22'53.3"S | 51°05'33.4"W | Female | Adult |
|  | Lung | 234.7 | 1.96 |  |  |  |  |  |
|  | Spleen | 1960.3 | 1.91 |  |  |  |  |  |
| *Patagioenas picazuro* | Liver | 905.0 | 1.93 | Winter/2023 | N/A | N/A | Female | Adult |
|  | Lung | 494.5 | 1.94 |  |  |  |  |  |
| *Didelphis albiventris* | Heart | 148.9 | 1.41 | Winter/2023 | 23°19'00.9"S | 51°11'25.3"W | Male | Adult |
|  | Liver | 788.0 | 2.05 |  |  |  |  |  |
|  | Lung | 348.3 | 1.93 |  |  |  |  |  |
|  | Spleen | 29.1 | 1.93 |  |  |  |  |  |
| *Dasypus novemcinctus* | Heart | 75.0 | 1.97 | Winter/2023 | N/A | N/A | Male | Adult |
|  | Liver | 2559.3 | 1.93 |  |  |  |  |  |
|  | Lung | 246.6 | 1.94 |  |  |  |  |  |
|  | Spleen | 1129.8 | 1.91 |  |  |  |  |  |
| *Didelphis albiventris* | Heart | 80.2 | 2.00 | Summer/2023 | N/A | N/A | Male | Juvenile |
|  | Liver | 327.5 | 1.99 |  |  |  |  |  |
|  | Lung | 409.4 | 1.95 |  |  |  |  |  |
|  | Spleen | 1976.4 | 1.97 |  |  |  |  |  |
| *Didelphis albiventris* | Heart | 317.8 | 1.99 | Winter/2023 | N/A | N/A | Male | Juvenile |
|  | Lung | 2696.5 | 1.95 |  |  |  |  |  |
|  | Spleen | 1934.5 | 1.97 |  |  |  |  |  |
| *Didelphis albiventris* | Heart | 62.4 | 2.04 | Winter/2023 | 23°21'16.6"S | 51°11'54.3"W | Male | Juvenile |
|  | Lung | 2009.2 | 1.96 |  |  |  |  |  |
|  | Spleen | 321.1 | 1.91 |  |  |  |  |  |
| *Thraupis sayaca* | Liver | 2404.3 | 2.03 | Winter/2023 | 23°19'41.9"S | 51°11'57.8"W | Male | Adult |

**Supplementary Table 2. Sanger sequencing of the qPCR-positive amplicons (~152-174 bp).***

| **Sample ID** | **Common name** | **Species detected (qPCR)** | **Amplicon sequence (5’→3’)** | **Size (bp)** | **Top BLAST hit (species/accession/identity %)** | **Notes on Sanger sequencing** |
| --- | --- | --- | --- | --- | --- | --- |
| *Colaptes melanochloros* | Green-barred woodpecker | *S. schenckii* | CGTCTGAGCGTCTACTTCAACGAGGTTTGTTTTTTGGTTTCCCGACCGACTTTGGTGTCACGTCCCACCGTTTGGCACCAGTCTAACCTAACCATTTTGATAGGCCTCTGGCAACAAGTACGTCCCCCGTGCCGTCCTCGTCGATCTCGAGCCTGGTACCATGGATGCCGTCC | 173 | *S. schenckii*/ AM116924/ 99.42% | Clear chromatogram, single-species profile (*i.e.*, no mixed peaks). |
| *Columbina picui* | Picui ground dove | *S. brasiliensis + S. globosa* | CGTCTGAGCGTCTACTTCAACGAGGTTTGTTTTTGGTTTCCCGATCGGCTTTGCTTTGGCCCTAGTCTAACCATTTTGATAGGCCTCTGGCAACAAGTACGTCCCCCGTGCCGTCCTGGTCGATCTCGAGCCCGGTACCATGGATGCCGTCC | 152 | *S. brasiliensis*/ OP545816/ 100.00%;  *S. globosa/* MG953931/ 98.73% | Conserved sites clean, ambiguous bases at divergent positions, supporting coinfection findings. |
| *Crypturellus tataupa* | Tataupa tinamou | *S. brasiliensis* | CGTCTGAGCGTCTACTTCAACGAGGTTTGTTTTTGGTTTCCCGATCGGCTTTGCTTTGGCCCTAGTCTAACCATTTTGATAGGCCTCTGGCAACAAGTACGTCCCCCGTGCCGTCCTGGTCGATCTCGAGCCCGGTACCATGGATGCCGTCC | 152 | *S. brasiliensis*/ OP545816/ 100.00% | Clear chromatogram, single-species profile (*i.e.*, no mixed peaks). |
| *Piaya cayana* | Squirrel cuckoo | *S. schenckii* | CGTCTGAGCGTCTACTTCAACGAGGTTTGTTTTTGGTTTCCCGACCGACTTTGGTGTCACGTCCCACCGTTTGGCACCAGTCTAACCTAACCATTTTGATAGGCCTCTGGCAACAAGTACGTCCCCCGTGCCGTCCTCGTCGATCTCGAGCCTGGTACCATGGATGCCGTCC | 172 | *S. schenckii*/ MK478787/ 100.00% | Clear chromatogram, single-species profile (*i.e.*, no mixed peaks). |
| *Selenidera maculirostris* | Spot-billed toucanet | *S. schenckii* | CGTCTGAGCGTCTACTTCAACGAGGTTTGTTTTTTGGTTTCCCGACCGGCTTCAGTGTCACGTCCCACCGTTTGGCACTAGTTTAACCTAACTATTTTGATAGGCCTCTGGCAACAAGTACGTCCCCCGTGCCGTCCTGGTCGATCTCGAGCCCGGTACCATGGATGCCGTCC | 173 | *S. schenckii*/ ON211480/ 100.00% | Clear chromatogram, single-species profile (*i.e.*, no mixed peaks). |
| *Dasyprocta* spp. | Agoutis | *S. globosa* | CGTCTGAGCGTCTACTTCAACGAGGTTTGTTTTTTGGTTTACCTACCGGCTTTGGTGTCACGTCACAGTTTTGGCACGATTCTAACAATTTTTGATAGGCCTCTGGCAACAAGTACGTCCCCCGTGCCGTCCTGGTCGATCTCGAGCCCGGTACCATGGATGCCGTCC | 168 | *S. globosa*/ KX881722/ 100% | Clear chromatogram, single-species profile (*i.e.*, no mixed peaks). |
| *Didelphis albiventris* | White-eared opossum | *S. schenckii* | CGTCTGAGCGTCTACTTCAACGAGGTTTGTTTTTGGTTTCCCGACCGACTTTGGTGTCACGTCCCACCGTTTGGCACCAGTCTAACCTAACCATTTTGATAGGCCTCTGGCAACAAGTACGTCCCCCGTGCCGTCCTCGTCGATCTCGAGCCTGGTACCATGGATGCCGTCC | 172 | *S. schenckii*/ MK478787/ 100.00% | Clear chromatogram, single-species profile (*i.e.*, no mixed peaks). |
| *Leopardus guttulus* | Southern tiger cat | *S. schenckii* | CGTCTGAGCGTCTACTTCAACGAGGTTTGTTTTTGGTTTCCCGACCGACTTTGGTGTCACGTCCCACCGTTTGGCACCATTCTAACCTAACCATTTTGATAGGCCTCTGGCAACAAGTACGTCCCCCGTGCCGTCCTCGTCGATCTCGAGCCTGGTACCATGGATGCCGTCC | 172 | *S. schenckii*/ MK478787/ 99.42% | Clear chromatogram, single-species profile (*i.e.*, no mixed peaks). |
| *Lepus europaeus* | European hare | *S. schenckii* | CGTCTGAGCGTCTACTTCAACGAGGTTTGTTTTTGGTTTCCCGACCGACTTTGGTGTCACGTCCCACCGTTTGGCACCAGTCTAACCTAACCATTTTGATAGGCCTCTGGCAACAAGTACGTCCCCCGTGCCGTCCTCGTCGATCTCGAGCCTGGTACCATGGATGCCGTCC | 172 | *S. schenckii*/ MK478787/ 100.00% | Clear chromatogram, single-species profile (*i.e.*, no mixed peaks). |
| *Lepus europaeus* | European hare | *S. schenckii* | CGTCTGAGCGTCTACTTCAACGAGGTTTGTTTTTTGGTTTCCCGACCGACTTCAGTGTCACGTCCCACCGTTTGGCACCAGTTCTAACCTAACCATTTTGATAGGCCTCTGGCAACAAGTACGTCCCCCGTGCCGTCCTCGTCGATCTCGAGCCTGGTACCATGGATGCCGTCC | 174 | *S. schenckii*/ MK478787/ 97.70% | Clear chromatogram, single-species profile (*i.e.*, no mixed peaks). |
| *Oxyrhopus* spp. | False coral snake | *S. schenckii + S. globosa* | CGTCTGAGCGTCTACTTCAACGAGGTTTGTTTTTGGTTTCCCGACCGACTTTGGTGTCACGTCCCACCGTTTGGCACCATTCTAACCTAACCATTTTGATAGGCCTCTGGCAACAAGTACGTCCCCCGTGCCGTCCTCGTCGATCTCGAGCCTGGTACCATGGATGCCGTCC | 172 | *S. schenckii*/ MK478787/ 99.42%;  *S. globosa/* MG953931/ 99.36% | Conserved sites clean, ambiguous bases at divergent positions, supporting coinfection findings. |

*The DNA sequences obtained from the qPCR-positive amplicons, which target a common intronic region in the *BT2* gene of *Sporothrix* (using primers Sporo-F and Sporo-R), were not submitted to GenBank (https://www.ncbi.nlm.nih.gov/genbank/) owing to their short fragment sizes (~152-174 bp), which are below the recommended minimum length for submission (≥ 200 bp). However, the complete amplicon sequence for each sample is provided in this table for reference.

**Supplementary Table 3.** Results of chi-square (χ^2^) and Fisher’s exact tests for associations between qPCR positivity and epidemiological variables in animal hosts.

| **Analyzed variable vs. qPCR result** | **Dimensions (Contingency)** | **χ^2^ statistic** | **Df** | ***p-*value (χ^2^)** | ***p-*value (Fisher’s - two-sided)** | **Significant association (α=0.05)** |
| --- | --- | --- | --- | --- | --- | --- |
| Animal class | 3×2 | 0.07080 | 2 | 0.9652 | — | No |
| Animal sex | 3×2 | 4.326 | 2 | 0.1150 | — | No |
| Development stage | 4×2 | 4.832 | 3 | 0.1845 | — | No |
| Proximity to urban center | 2×2 | 4.095 | 1 | 0.0430 | 0.0790 | No¹ |
| **Road type** | **4×2** | **9.640** | **3** | **0.0219** | **—** | **Yes** |
| Season | 4×2 | 1.598 | 3 | 0.6599 | — | No |
| Presence of shoulder | 2×2 | 0.000 | 1 | 1 | 1 | No¹ |
| Presence of riparian forest | 2×2 | 0.1268 | 1 | 0.7218 | 1 | No¹ |
| Conservation status (IUCN) | 2×2 | 1.036 | 1 | 0.3088 | 0.3584 | No¹ |
| Postmortem condition | 5×2 | 2.011 | 4 | 0.7338 | — | No |
| **Interaction with domestic animals** | **2×2** | **5.809** | **1** | **0.0159** | **0.0322** | **Yes¹** |

¹For 2×2 tables with low expected values, the conclusion is based on Fisher’s exact test, which is more accurate under these conditions. IUCN = International Union for Conservation of Nature.

**Supplementary Table 4.** Distribution of β-tubulin haplotypes, GenBank accession numbers, and species identification for the clinical and environmental *Sporothrix* isolates analyzed.

| **Isolate code / Sequence ID** | **Species** | **GenBank** | **Haplotype** |
| --- | --- | --- | --- |
| IPEC 17943 | *Sporothrix brasiliensis* | AM116935 | H1 |
| PR-538 (*Crypturellus tataupa*) | *Sporothrix brasiliensis* | - | H1 |
| IPEC 22582 | *Sporothrix brasiliensis* | AM116956 | H1 |
| IPEC 16490 | *Sporothrix brasiliensis* | AM116946 | H1 |
| IPEC 15572 | *Sporothrix brasiliensis* | AM116955 | H1 |
| CBS 130114 (IHEM 15503) | *Sporothrix schenckii* | AM116930 | H2 |
| IHEM 15486 | *Sporothrix schenckii* | AM116929 | H2 |
| PR-445 (*Piaya cayana*) | *Sporothrix schenckii* | - | H3 |
| Rural (*Didelphis albiventris*) | *Sporothrix schenckii* | - | H3 |
| PR-445 (*Lepus europaeus*) | *Sporothrix schenckii* | - | H3 |
| PR-445 (*Leopardus guttulus*) | *Sporothrix schenckii* | - | H4 |
| CBS 359.36 | *Sporothrix schenckii* | AM116911 | H5 |
| Rural (*Selenidera maculirostris*) | *Sporothrix schenckii* | - | H5 |
| CMW 7614 | *Sporothrix schenckii* | AY280477 | H6 |
| CMW 7615 | *Sporothrix schenckii* | AY280478 | H6 |
| CMW 7612 | *Sporothrix schenckii* | AY280476 | H6 |
| CMW 5681 | *Sporothrix schenckii* | EF139107 | H6 |
| FMR 8677 | *Sporothrix schenckii* | AM116915 | H7 |
| BR-376 (*Colaptes melanochloros*) | *Sporothrix schenckii* | - | H8 |
| Urban (*Lepus europaeus*) | *Sporothrix schenckii* | - | H9 |
| CBS 130105 | *Sporothrix globosa* | AM116964 | H10 |
| CBS 120340 | *Sporothrix globosa* | AM116966 | H10 |
| CBS 130116 | *Sporothrix globosa* | AM116962 | H10 |
| CBS 130115 | *Sporothrix globosa* | AM116963 | H10 |
| CBS 129724 | *Sporothrix globosa* | KC113238 | H10 |
| Urban (*Dasyprocta* spp.) | *Sporothrix globosa* | - | H10 |
| CBS 130104 | *Sporothrix globosa* | AM116959 | H11 |
| ATCC 18616 | *Sporothrix luriei* | AM747289 | H12 |
| CMW 7618 | *Sporothrix humicola* | EF139100 | H13 |
| CMW 14541 | *Sporothrix stylites* | EF139094 | H14 |
| CMW 14543 | *Sporothrix stylites* | EF139096 | H14 |
| CMW 20677 | *Sporothrix palmiculminata* | DQ821543 | H15 |
| CBS 139891 | *Sporothrix chilensis* | KP711813 | H16 |
| CBS 139890 | *Sporothrix chilensis* | KP711814 | H16 |
| CBS 147636 | *Sporothrix davidellisii* | MK380725 | H17 |
| CBS 120341 | *Sporothrix mexicana* | AM498344 | H18 |
| CBS 15087 | *Sporothrix pallida* | EF139109 | H19 |
| CBS 13156 | *Sporothrix pallida* | EF139110 | H20 |
| CBS 55374 | *Sporothrix dimorphospora* | AY495439 | H21 |
| CBS 125442 | *Sporothrix dimorphospora* | FN547379 | H22 |
| CMW 13016 | *Sporothrix dentifunda* | AY495445 | H23 |
| CMW 13017 | *Sporothrix dentifunda* | AY495446 | H23 |
| CMW 12535 | *Sporothrix inflata* | AY495442 | H24 |
| CMW 12527 | *Sporothrix inflata* | AY495437 | H24 |
| CMW 11193 | *Sporothrix stenoceras* | AY280475 | H25 |
| CMW 3202 | *Sporothrix stenoceras* | DQ296074 | H25 |
| CMW 23060 | *Sporothrix variecibatus* | DQ821573 | H26 |
| CMW 23051 | *Sporothrix variecibatus* | DQ821539 | H26 |
| CMW 872 | *Sporothrix splendens* | DQ836011 | H27 |
| CMW 1103 | *Sporothrix protearum* | DQ316165 | H28 |
| CMW 1107 | *Sporothrix protearum* | DQ316163 | H28 |
| CMW 1104 | *Sporothrix africana* | DQ316162 | H29 |
| CMW 1822 | *Sporothrix africana* | DQ316159 | H30 |
| CMW 19362 | *Sporothrix aurorae* | DQ396800 | H31 |
| CMW 1468 | *Sporothrix abietina* | EU977484 | H32 |
| CMW 8281 | *Sporothrix fusiformis* | AY280462 | H33 |
| CMW 8285 | *Sporothrix fusiformis* | AY280463 | H33 |
| CMW 10563 | *Sporothrix lunata* | AY280466 | H34 |
| CMW 10564 | *Sporothrix lunata* | AY280467 | H34 |
| CBS 474.91 | *Sporothrix bragantina* | FN547387 | H35 |
| CBS 430.92 | *Sporothrix bragantina* | FN547386 | H36 |
| CMW 20676 | *Sporothrix phasma* | DQ821541 | H37 |
| CMW 20686 | *Sporothrix phasma* | DQ316185 | H38 |
| CMW 650 | *Ophiostoma nigricarpum* | AY280479 | H39 |
| CMW 651 | *Ophiostoma nigricarpum* | AY280480 | H39 |
| FMR 9338 | *Sporothrix brunneoviolacea* | FN547385 | H40 |
| CMW 18600 | *Hawksworthiomyces lignivorus* | EF139104 | H41 |
| CMW 18601 | *Hawksworthiomyces lignivorus* | EF139105 | H41 |
| CMW 18599 | *Hawksworthiomyces lignivorus* | EF139103 | H41 |
